# Labeling of Amine-Modified Material Surfaces with PFB-Fluorescein

**DOI:** 10.2147/NSA.S576032

**Published:** 2026-03-25

**Authors:** Kaja Jaskot, Angelika Maria Mielcarek, Łukasz Popenda, Ahmet Kertmen, Olena Ivashchenko, Bartosz F Grześkowiak, Agnieszka Fedoruk-Wyszomirska, Kamilla Bąkowska-Żywicka, Agata Tyczewska, Jan Barciszewski, Patrick M Perrigue

**Affiliations:** 1 NanoBioMedical Centre, Adam Mickiewicz University, Poznań, Poland; 2 Faculty of Chemistry, Adam Mickiewicz University, Poznań, Poland; 3 Faculty of Physics and Astronomy, Adam Mickiewicz University, Poznań, Poland; 4 Institute of Human Genetics, Polish Academy of Sciences, Poznań, Poland; 5 Institute of Bioorganic Chemistry, Polish Academy of Sciences, Poznań, Poland

**Keywords:** fluorescein, PFB-F, nanoparticles, material surface, conjugate chemistry, *C. elegans*

## Abstract

**Introduction:**

Fluorescent labeling is a widely used method for visualizing and tracking materials. 5-(Pentafluorobenzoylamino) fluorescein (PFB-F) is a dye in which the pentafluorobenzoylamino group substituent functions as a reactive linker. While PFB-F has been used for labeling biomolecules, its application in labeling surfaces has not been explored yet. Here, we report straightforward method for attaching PFB-F to amine-modified material surfaces requiring only dimethyl sulfoxide (DMSO) solvent and room temperature (RT).

**Methods:**

We conducted nuclear magnetic resonance (NMR) and thin layer chromatography (TLC) analysis on a mock reaction involving PFB-F and *N*-butylamine to evaluate the process of attachment via the PFB linker. PFB-F was also reacted with poly-L-lysine to evaluate conjugation to polymers bearing primary amine groups. Silica nanoparticles (SiNPs) labeled with PFB-F were administered to nematode worms, followed by confocal fluorescence microscopy.

**Results:**

NMR and TLC analysis confirmed covalent bonding between PFB-F and primary amines via nucleophilic aromatic substitution (S_N_Ar) involving displacement of at least one fluorine atom. PFB-F was successfully conjugated to poly-L-lysine, demonstrating efficient reaction with polymers containing primary amine groups. SiNPs labeled with PFB-F were ingested by nematode worms and successfully tracked in vivo.

**Discussion:**

These results demonstrate that PFB-F is a versatile and effective reagent for stable surface labeling. This study paves the way for broader applications of PFB-F in material design.

## Introduction

The development of efficient fluorescent labeling strategies is crucial for advancing biomedical sensors and imaging technology.^1^ Fluorescein is a widely used fluorescent label due to its strong excitation and emission characteristics.^2^ A well-established strategy for the conjugation of fluorescein is using fluorescein isothiocyanate (FITC), which has been used to label proteins, antibodies, and nanocarriers.^3^,^4^ In past years, numerous other strategies have been developed. Click chemistry which use alkynes and azides, offer chemoselective functional groups for the attachment of fluorescein.^5^,^6^ PFB-F represents an alternative conjugation strategy that is well suited for use in organic solvents and does not require catalysts. PFB-F can react with thiol groups in proteins.^7^ It has also been used as a selective substrate to measure glutathione concentration and glutathione S-transferase activity.^8^ While chemically distinct from the PFB linker, pentafluorobenzene by itself is a well-known reagent for crosslinking amine groups.^9^ However, the use of the PFB linker as a versatile conjugation method for fluorescein is underexplored.

The conjugation of fluorescein with nanoparticles enables a substantial expansion of its applications by improving signal stability, bioavailability, and the biological selectivity of diagnostic and therapeutic systems.^10^ Fluorescein can be covalently attached to the functionalized surface of nanoparticles or encapsulated within their interior. Such approaches reduce fluorescein photobleaching, limit its nonspecific diffusion in biological environments, and allow for controlled localization of the fluorescent signal.^11^ Fluorescein-conjugated nanoparticles are used for cell and tissue imaging, tracking endocytic processes, and visualizing pathological changes. Their application is particularly important in intracellular imaging, where nanocarriers facilitate the delivery of the fluorophore to specific organelles.^12^ When combined with targeting ligands (e.g., peptides or antibodies), high specificity toward selected cell types, including cancer cells, can be achieved.^13^ Fluorescein-conjugated nanoparticles are also employed in systems in which changes in fluorescence intensity respond to biological stimuli such as pH, enzymatic activity, or the presence of specific biomolecules.^14^ Such systems are especially promising for molecular diagnostics and real-time monitoring of disease-related processes.

SiNPs are widely used in biomedicine due to their high colloidal stability and tunable surface chemistry.^15^,^16^ Recently, PFB-based conjugation chemistry was applied to modifying the surface of SiNPs. For example, PFB-porphyrin was immobilized onto amine-modified magnetic silica particles.^17^ Similarly, magnetic SiNPs functionalized with PFB-phthalocyanine have been synthesized using the same mechanism.^18^ These modifications were performed under reaction conditions that favor reactivity with primary amines. This strategy holds significant potential for labeling other types of material surfaces. The use of PFB-F for labeling amine-modified materials has not yet been systematically investigated. We address this gap by evaluating the conjugation efficiency and its stability, offering new insights into its utility as a reactive fluorophore for labeling advanced materials in bioimaging applications.

*Caenorhabditis elegans* (*C. elegans*) is a model organism used for studying the biological fate and toxicity of nanoparticles.^19^ Owing to its transparent and thin body, fluorescence from labeled SiNPs can be tracked in vivo.^20^ *C. elegans* can readily ingest these SiNPs, allowing their passage through the intestinal tract to be directly observed. We employed a dual-labeling strategy in which SiNPs contained a rhodamine B-labeled core and a PFB-F-labeled surface, enabling colocalization analysis during imaging. Our results demonstrate that PFB-F is an effective and stable fluorophore for the surface labeling of advanced materials, enabling their precise tracking in biological environments.

## Materials and Methods

### Preparation of Stock Chemicals

5-(Pentafluorobenzoylamino) fluorescein (PFB-F, AG002KAM, Chemat, Gdańsk, Poland) and fluorescein (F2456, Sigma, St. Louis, MO, USA) were dissolved in dimethyl sulfoxide (DMSO, D2438, Sigma, St. Louis, MO, USA) to prepare 10 mg/mL stock solutions. All stock solutions were stored at −20°C until use.

### Nuclear Magnetic Resonance

NMR measurements were conducted using an Agilent DD2 600 spectrometer (Agilent Technologies, Santa Clara, CA, USA) equipped with a OneNMR probe at a temperature of 25 °C. The experiments were performed in DMSO-*d*_6_ with an approximate PFB-F concentration of 2 mg/mL. Following the acquisition of PFB-F spectra, an equimolar amount of *N*-butylamine was added directly to the NMR tube. Data were collected using Agilent’s standard pulse sequences and acquisition parameters. All ^19^F NMR spectra were referenced to CFCl_3_ (δ = 0.0 ppm) as an internal standard.

### Thin Layer Chromatography

TLC was carried out by applying 1 µL of each reaction mixture onto silica gel plates (1.00390.0001, Sigma-Aldrich Canada Co., Canada), followed by air drying. The chromatographic separation was performed using a 1:1:1 (v/v/v) mixture of 1-butanol, methanol, and water as the mobile phase. Plates were removed from the chamber when the solvent front had reached approximately three-quarters of the total plate height. After drying, fluorescence analysis was conducted using a Bio-Rad PharosFX™ Plus Molecular Imager with excitation at 488 nm.

### Coverslips

Glass coverslips (16 × 16 mm, Comex, Wrocław, Poland) were rinsed with ethanol and allowed to air dry. The coverslips were then transferred to a 6-well format cell culture plate and then coated by applying a thin layer of poly-L-lysine (P8920, Sigma, St. Louis, MO, USA) for 1 h at room temperature. After incubation, the coverslips were rinsed with Milli-Q water to remove any unbound poly-L-lysine. Subsequently, the coverslips were covered in a solution of PFB-F or fluorescein, prepared by dissolving 5 µL of stock in 1 mL of DMSO, and incubated for 2 h. Following incubation, they were handled using forceps and gently washed by dunking them three times into a 500 mL beaker filled with Milli-Q water. Fluorescence signal was detected using the Pharos FX Plus imaging system (Bio-Rad, Hercules, CA, USA).

### Labeling of SiNPs

SiNPs with a diameter of 500 nm, functionalized with surface amino groups (PSI-0.5NH2) and those with rhodamine B in their core (PSI-R0.5NH2) were obtained from Kisker Biotech GmbH & Co. KG (Steinfurt, Germany). To prepare SiNPs for labeling, 50 µL of SiNPs were added to a 1.5 mL Eppendorf tube and washed two times with 1 mL of DMSO by centrifugation at 12,000 rpm for 10 min., discarding the supernatant after each wash. The SiNPs were then resuspended in 1 mL of DMSO, and 5 µL of PFB-F or fluorescein stock solution was added as a negative control. The mixture was incubated at RT for overnight. After incubation, the samples were washed three times with 1 mL of PBS (524650, Millipore, Burlington, MA, USA) to remove unbound fluorophore. The final pellet of labeled SiNPs was resuspended in 1 mL of PBS.

### Characterization of SiNPs

For imaging flow cytometry analysis, a 2 µL aliquot of the labeled SiNPs suspension was diluted in 200 µL of PBS. The samples were analyzed using a FlowSight Imaging Flow Cytometer with a 488 nm wavelength laser (Amnis, Luminex Corporation, Seattle, WA, USA). Histograms and images were generated using IDEAS software.^21^ For ζ-potential measurements, freshly prepared SiNPs were first sonicated for 1 min with 10s pulse intervals at 25% amplitude using a Branson Sonifier (Branson Ultrasonics, Danbury, CT, USA). Subsequently, 1 µL of the nanoparticle suspension was diluted in 999 µL of Milli-Q water, and the ζ-potential was measured using a Zetasizer Nano ZS (Malvern Panalytical, Malvern, UK). The reported values in the text represent the mean ± standard deviation from three replicates. SiNPs were further characterized using a JEOL 7001 F scanning electron microscope. The SiNPs were mounted on carbon tape and gold sputtered. The images were taken using SEI detector, 15 kV accelerating voltage, WD 10 mm and magnification × 50,000. For each condition, the diameter of SiNPs was determined by importing micrographs into ImageJ and measuring individual particle diameters with the line tool. The values are reported as the mean diameter ± standard deviation, calculated from 150 individual measurements per condition.

### Fluorescence Imaging of C. elegans

Research involving *C. elegans* does not require animal ethics committee approval under institutional or national guidelines. Under the European Union Directive 2010/63/EU, only live non-human vertebrates and cephalopods are regulated. All other invertebrates, including nematodes such as *C. elegans*, lie outside the directive’s scope. All procedures were performed with attention to minimizing stress and ensuring responsible and respectful handling of the animals throughout the study. Twenty-five *C. elegans* adult worms, variety Bristol, strain N2, were transferred onto freshly prepared NG2% plates seeded with *E. coli* OP50 strain mixed with SiNPs at a final concentration of 50 ng/mL and grown at 20 °C. The worms were fed *E. coli* with SiNPs for the 4 days. Next, the worms were transferred to fresh NG2% plates without *E. coli* and kept for another 6 h. Four worms were collected and washed in M9 buffer to prevent SiNPs contamination on the cuticle. Subsequently the worms were fixed using 4% paraformaldehyde for 15 min. After fixation worms were transferred to a glass slide covered with 1% agar, immobilized with SlowFade^®^ Gold Antifade Reagent (Life Technologies) and topped with a cover glass. Sequentially scanned Z-stack images were acquired using the tail scan at an excitation/emission wavelength of 490/500–560 nm for fluorescein (green), and 558/565–650 nm for rhodamine B (red), using a Leica STELLARIS confocal microscope with a white laser and an HC PL APO CS2 100×/1.4 oil-immersion objective. TAU contrast was used to obtain an image of the fluorescence average arrival time. Acquisition and image processing were carried out using Leica LAS AF 4.6.1 software. Z-projection of images was generated from Z-stacks using the max-intensity projection and the 3D viewer in LAS X software.

To quantify the fluorescence ratio of SiNPs, images were split into individual color channels using Fiji software.^22^ The rhodamine B (red) channel was automatically thresholded, and SiNPs were identified using the “Analyze Particles” feature. The same regions of interest (ROI) were overlayed to the fluorescein (green). Fluorescence intensity values for each ROI were measured using the “Measure” feature. “Raw Integrated Density” values were plotted on the graph with a linear regression statistic using Origin.

## Results and Discussion

### Reaction Conditions with Primary Amines

**Figure 1:**
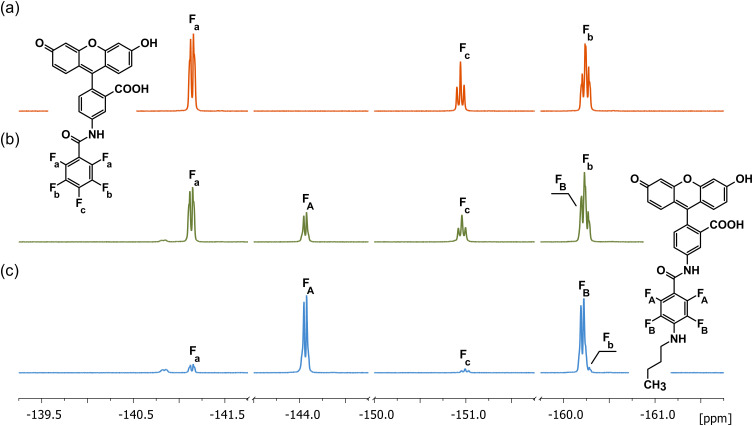
^19^F NMR study of the substitution reaction of PFB-F with *N*-butylamine (DMSO-*d*_6_, 25 °C). Selected regions of the spectra present signals corresponding to fluorine atoms in different ring positions, illustrating spectral changes during the substitution reaction. (**a**) PFB-F (**b**) ~30% substitution with *N*-butylamine at the *para* position (**c**) ~90% completion of the reaction. Labeling of the signals corresponds to the atom labels in the structures.

To assess the reactivity of PFB-F with amine groups, we analyzed its reaction with *N*-butylamine using ^19^F NMR spectroscopy. The reaction progress was monitored in DMSO-*d*_6_ at room temperature. The initial spectrum (Figure 1), corresponding to PFB-F, exhibited fluorine resonances of the pentafluorobenzyl moiety at −141.13 ppm (*ortho*), −160.23 ppm (*meta*), and −150.93 ppm (*para*). Following the addition of *N*-butylamine, the gradual disappearance of these signals, along with the appearance of new peaks at −144.06 ppm (*ortho*) and −160.21 ppm (*meta*), provided clear evidence for substitution. The reaction proceeds via a S_N_Ar mechanism, with concomitant HF elimination. The observed regioselectivity is consistent with previously reported reactivity of pentafluorophenyl derivatives, where electronic effects and steric accessibility favor substitution at the para position.^23–26^

**Figure 2:**
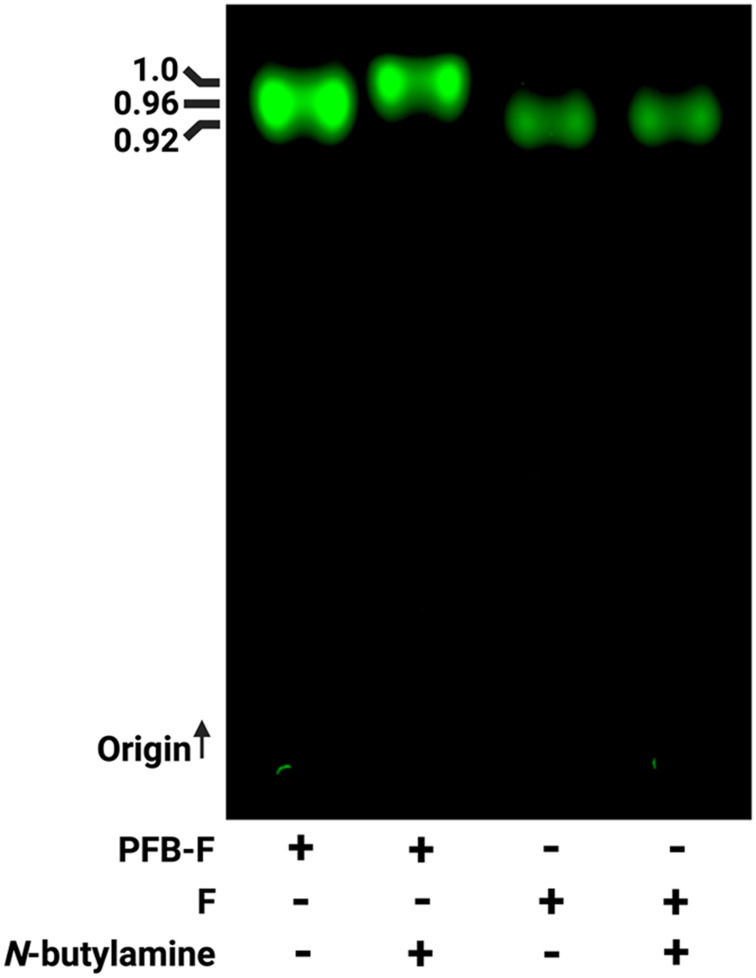
TLC analysis of PFB-F, fluorescein (F), and their respective mixtures with N-butylamine. “+” and “−” denote the presence or absence of the indicated compound(s) in each lane. The corresponding retention factor (Rf) values are indicated. The origin and solvent front are labeled for reference, with the arrow ↑ indicating the direction of TLC development.

We also performed TLC as additional proof of the reaction product between PFB-F and *N*-butylamine (Figure 2). TLC showed that both fluorescein and PFB-F migrate with distinct differences in retention: (Rf) = 0.92 for fluorescein and 0.96 respectfully. With *N*-butylamine, fluorescein retained the same (Rf) = 0.92, indicating no reactivity. However, under the same reaction conditions, the fluorescence signal from PFB-F shifted to the solvent front with a new (Rf) = 1.0. This observation is consistent with the formation of a new derivative different from the starting materials and that PFB-F can be conjugated to a primary amine containing molecule under mild conditions, requiring only DMSO and room temperature.

### Fluorescent Labeling of Amine-Modified Material Surfaces

**Figure 3:**
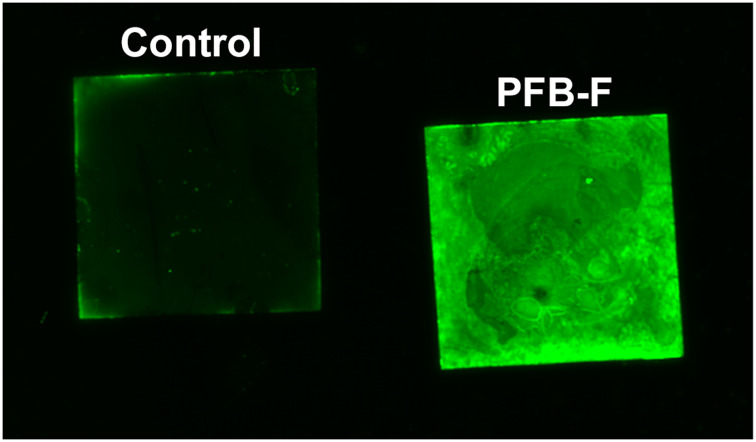
Conjugation of PFB-F to poly-L-lysine-coated glass coverslips. Coverslips were first coated with poly-L-lysine and then incubated with either fluorescein (control, left) or PFB-F (right). Samples were excited at 488 nm and imaged under the same condition.

To evaluate PFB-F for covalent attachment onto amine-functionalized material surfaces, we initially tested its reactivity with glass coverslips coated with poly-L-lysine (Figure 3). The glass coverslips served as a solid support to anchor poly-L-lysine, which binds well to the glass surface while leaving some amine groups available to react. This setup also allows for the assessment whether the fluorescent label has been immobilized. The coverslips coated with poly-L-lysine were submerged in a DMSO solution containing PFB-F or fluorescein for 2 h at RT. Fluorescence imaging revealed a strong and uniformly distributed green signal across the surface of the coverslip, indicating that PFB-F had reacted with poly-L-lysine. In contrast, the fluorescein control exhibited only a background fluorescence.

**Figure 4:**
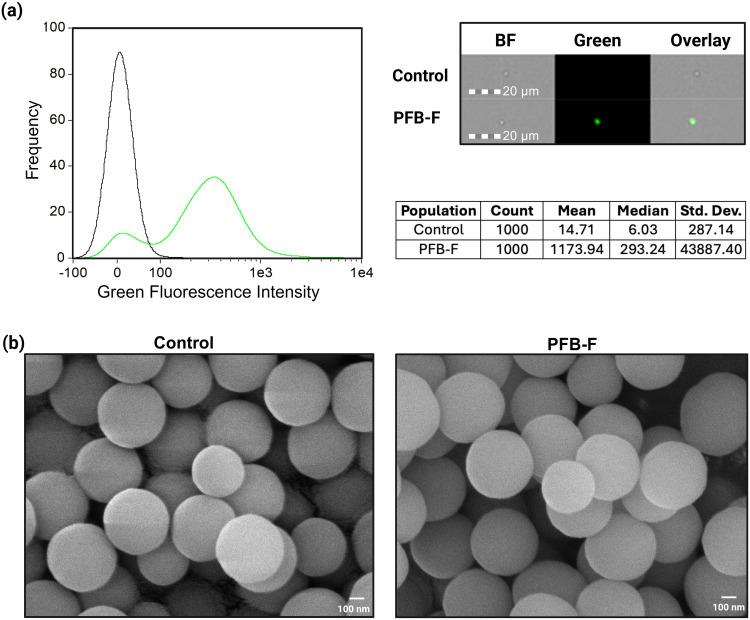
(**a**) IFC histograms showing increased green fluorescence in PFB-F labeled SiNPs (green line) compared to fluorescein used as a control (black line). To the right of the histogram are representative IFC images that confirm surface-localized fluorescence and the statistics table for the histogram. BF = brightfield channel, Green = fluorescein channel and their overlay. (**b**) SEM images of 500 nm amine-modified SiNPs before (Control) and after PFB-F labeling (PFB-F).

We next tested the reactivity of PFB-F with 500 nm amine-functionalized SiNPs (500SiNP-NH_2_) to produce 500SiNP-NH-PFB-F. The labeling reaction was carried out in DMSO for 18 h at RT, followed by washing steps to remove unreacted dye. Imaging flow cytometry (IFC) was used to confirm the attachment of PFB-F (Figure 4a). To rule out any nonspecific adsorption, a control 500SiNP-NH_2_ sample was prepared with fluorescein. This negative control sample showed no fluorescence, suggesting that PFB-F must bind covalently with surface amines. The IFC images show that the fluorescent signal overlaps with the brightfield images, further confirming that PFB-F is localized on the surface. ζ-potential measurements were used to evaluate changes in surface charge following modification with PFB-F. A ζ-potential of + 18 ± 4 mV was measured for 500SiNP-NH_2_, consistent with the presence of surface amine groups. After reaction with PFB-F, the ζ-potential shifted to − 8 ± 1 mV for 500SiNP-NH-PFB-F. This decrease and reversal in surface charge indicates successful modification of the amine groups, resulting in consumption of the positively charged amines. Scanning electron micrographs of these samples revealed that SiNPs retain their size, smooth surfaces, and monodispersity after modification from 500SiNP-NH_2_ to 500SiNP-NH-PFB-F, indicating that the chemical reaction does not affect structural integrity. (Figure 4b). The average diameter of 500SiNP-NH_2_ and 500SiNP-NH-PFB-F were 480 ± 39 nm and 478 ± 46 nm, respectively. Altogether, these results demonstrate that the PFB linker enables specific covalent immobilization of fluorescein on amine-modified material surfaces via S_N_Ar.

### In vivo Tracking of PFB-F-Labeled SiNPs in C. elegans

**Figure 5:**
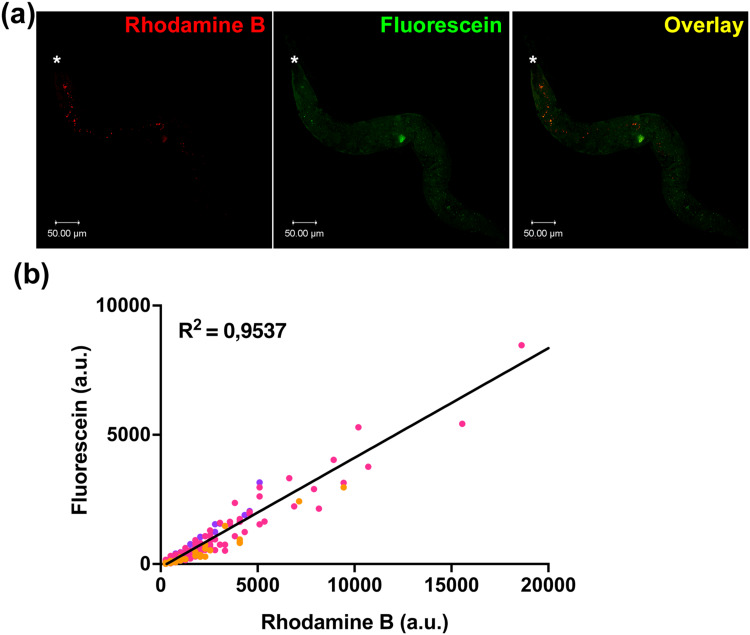
(**a**) Confocal fluorescence microscopy images of adult *C. elegans* fed with *E. coli* OP50 mixed with 500RhodB⁺SiNP-NH-PFB-F. Panels show fluorescence channels corresponding to rhodamine B, fluorescein, and their overlay. The asterisk (*) denotes the pharyngeal region of the worm. (**b**) Quantitative analysis of fluorescence co-localization in three worms. Each data point represents a region of interest containing rhodamine B signal plotted against fluorescein intensity (a.u. = arbitrary units). The data points for Worm 1 (pink, N = 62), Worm 2 (Orange, N = 252), and Worm 3 (magenta, N = 90) are shown. The linear regression line and corresponding *R^2^* value are indicated on the plot.

To further demonstrate the versatility of PFB-F as a labeling reagent, we applied it to SiNPs for in vivo tracking studies. We used 500 nm SiNP-NH_2_ particles with a rhodamine B-labeled core (500RhodB⁺SiNP-NH_2_) which were further labeled with PFB-F to produce 500RhodB⁺ SiNP-NH-PFB-F. Feeding of SiNPs in *C. elegans* has been previously described.^27–29^ We fed worms with 500RhodB⁺ SiNP-NH-PFB-F mixed with the OP50 E. coli strain, the standard bacterial food source used in laboratory maintenance. (Figure 5a). When worms were illuminated with excitation laser wavelengths specific for rhodamine B and fluorescein, control worms exhibited only background fluorescence. In contrast, worms fed with 500RhodB⁺ SiNP-NH-PFB-F displayed fluorescent signals localized in the gut, extending from the pharynx to the intestinal region. Further analysis of ingested 500RhodB⁺ SiNP-NH-PFB-F in worms showed a strong correlation overlap between the rhodamine B and fluorescein signals, with a linear regression R^2^ value of 0.9537 (Figure 5b). These findings validate PFB-F as a fluorescent tag for tracking SiNPs in vivo.

## Conclusion

We demonstrate that PFB-F serves as a versatile and effective reagent for covalent fluorescent labeling of primary amine-containing molecules and material surfaces. The reaction proceeds under mild conditions and enables efficient conjugation to poly-L-lysine and amine-functionalized SiNPs. The resulting fluorescent labels are stably associated with the modified materials and retain strong signal under in vitro conditions, supporting the utility of this method for surface-specific labeling. In vivo experiments further demonstrated that PFB-F-labeled SiNPs can be visualized in an intact organism, with dual-labeling confirming that surface and core components remain associated.

## Data Availability

Data are available upon request from the correspondence author.
